# Whole-genome resequencing revealed the genetic diversity and body weight-related genes of Gannan yak

**DOI:** 10.3389/fvets.2025.1540523

**Published:** 2025-06-24

**Authors:** Minghao Zhang, Chun Huang, Qi Bao, Tong Wang, Xiaoming Ma, Guangyao Meng, Xuelan Zhou, Qingbo Zheng, Min Chu, Chunnian Liang, Xian Guo, Pengjia Bao, Ping Yan

**Affiliations:** ^1^Key Laboratory of Animal Genetics and Breeding on Tibetan Plateau, Ministry of Agriculture and Rural Affairs, Lanzhou Institute of Husbandry and Pharmaceutical Sciences, Chinese Academy of Agricultural Sciences, Lanzhou, China; ^2^Key Laboratory of Yak Breeding in Gansu Province, Lanzhou Institute of Husbandry and Pharmaceutical Sciences, Chinese Academy of Agricultural Sciences, Lanzhou, China; ^3^Shenzhen Branch, Guangdong Laboratory of Lingnan Modern Agriculture, Key Laboratory of Livestock and Poultry Multi-Omics of MARA, Agricultural Genomics Institute at Shenzhen, Chinese Academy of Agricultural Sciences, Shenzhen, China

**Keywords:** Gannan yak, body weight, genetic diversity, genome-wide association study, genome-wide scanning

## Abstract

Body weight (BW) is a crucial indicator of animal growth and development, significantly influencing animal husbandry practices. Previous research has identified several genes associated with BW in certain yak breeds. However, the genetic basis of BW in Gannan yaks has not been reported. In this study, 309 yaks from six breeds across five provinces in China were sampled. This collection included 247 54-month-old female Gannan yaks, along with 20 Xizang yaks, 15 Muli yaks, 10 Pamir yaks, 9 Bazhou yaks, and 8 Zhongdian yaks. Body weight measurements were recorded for the Gannan yaks. Initial analyses of runs of homozygosity (ROH), nucleotide diversity, and linkage disequilibrium (LD) decay among the six yak breeds revealed that Gannan yaks exhibited the lowest ROH, the highest nucleotide diversity, and the fastest LD decay, indicating rich genetic diversity. Subsequently, a genome-wide association study (GWAS) identified 19 BW-related genes in the Gannan yaks, with *PMAIP1*, *GABBR1*, *LRPPRC*, and *PPP1R11* identified as key genes. Genome-wide scanning of Group 1 and Group 2 (detailed in section 2.4) identified 90 genes, and Gene Ontology (GO) analysis highlighted *FGF2*, *SHH*, and *WNT11* as significantly associated with growth, development, and metabolism. Three overlapping genes were identified between GWAS and genome-wide scans. Further analyses, including nucleotide diversity, LD analysis of significant GWAS sites, allele frequency analysis, and SNP association studies, suggested that *DRC1* and *SELENOI* are novel candidate genes for BW in Gannan yaks. These findings provide a molecular foundation for the genetic improvement of Gannan yaks.

## 1 Introduction

Gannan yak, an ancient breed native to the Gannan Tibetan Autonomous Prefecture, is well-adapted to plateau environments and plays a vital role in the local economy (1). These yaks are known for their rich content of amino acids and trace elements in meat, highlighting their nutritional value. However, due to reliance on natural grassland grazing and the lack of scientific feeding and breeding practices, the breed has experienced degradation and decreased production performance. This has led to significant individual variations in BW among animals of the same age and gender.

Body weight is a critical growth trait directly reflecting the production performance of yaks and serving as an important indicator for meat production potential (2). Enhancing BW research is a key strategy to improve the productivity of Gannan yaks. Identifying essential functional genes associated with BW can significantly aid in the breeding and improvement of yak production performance. However, traditional crossbreeding efforts between yaks and cattle face challenges such as male sterility, chromosomal incompatibility, and genomic conflicts, limiting their effectiveness in improving yak breeds (3). Advances in DNA sequencing technology provide valuable tools for strengthening breeding efficiency (4). Since the first yak reference genome was sequenced in 2012 (5) and a chromosome-scale genome was published in 2020, researchers have gained new opportunities to conduct large-scale molecular breeding (6). Previous studies have identified BW-associated genes such as *MFSD4*, *LRRC37B*, and *NCAM2* in Maiwa yak through GWAS (2), and genes like *AHR*, *HPGDS*, *HSF1*, *SOX5*, *SOX6*, and *SOX8* in Ashidan yak through copy number variation analysis (CNV) (7–11). However, compared to livestock such as cattle, pig, and sheep, the breeding progress of yak has lagged behind. The Animal Quantitative Trait Loci (QTL) Database (Animal QTLdb) is a comprehensive repository that collects publicly available data on QTL, including phenotype and expression QTL (eQTL), GWAS, and CNV for livestock species. Among the extensively studied species, cattle, pigs, and sheep dominate, with 192,336 QTL records for cattle, 56,615 for pigs, and 5,058 for sheep, representing hundreds of base traits and trait variants curated from thousands of publications. In contrast, research on yak, particularly the Gannan yak, has significantly lagged behind in terms of QTL data and breeding advancements. Despite its economic and ecological importance in high-altitude regions, the breeding progress of Gannan yak remains limited. There is an urgent need to leverage molecular breeding techniques to identify key molecular markers associated with its growth traits.

In this study, we utilized a combination of GWAS, genome-wide scanning, and SNP association analyses to identify candidate genes associated with the BW trait in Gannan yaks. Such efforts would accelerate artificial selection, enhance productivity, and help preserve this valuable breed while bridging the gap in genomic research between yak and other livestock species.

## 2 Materials and methods

### 2.1 Sample collection and phenotypic measurement of Gannan yaks

**Figure 1:**
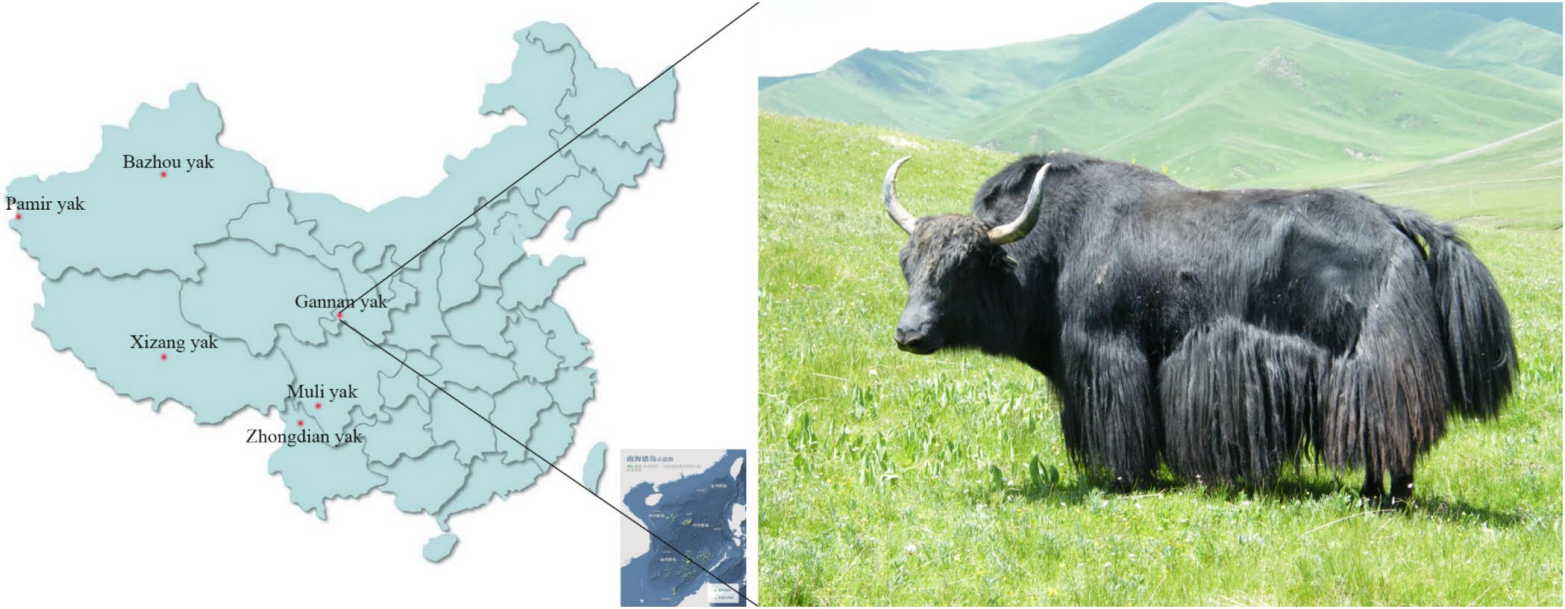
The distribution of six yak breeds employed in this study in China and the appearance of Gannan yak.

A total of 309 pasture-raised yaks were collected, all of them were adult domesticated yaks. They include 9 Bazhou yaks and 10 Pamir yaks, which were distributed in Xinjiang Uygur Autonomous Region of China. Twenty yaks in the Tibet Tibetan Autonomous Region of China, latter text is simply called Xizang yak; 15 Muli yaks in Sichuan Province, China; 8 Zhongdian yaks in Yunnan Province and 247 54-month-old female Gannan yaks in Gansu Province, China (Figure 1). Blood samples of 5 mL were obtained from the veins of each yak, transferred into EDTA anticoagulant tubes, and preserved at −20°C until subsequent analysis. The BW data (Table 1) of 247 Gannan yaks were collected in 54-month-olds in October 2023, and descriptive statistics were conducted using R (v4.3.3) language. All yak breeds not have pedigree records. The body weight of Gannan yaks was measured using a floor scale in the morning before fasting. The measurements were taken on two consecutive days, and the average value was calculated, expressed in kilograms (kg).

**Table 1:** Descriptive statistics of BW of Gannan yaks.

Item	Number	Mean	SD	Max	Min	SE
All	247	218.0	34.6	337.1	162.2	2.2
Group 1	20	287.0	16.6	337.1	270.3	3.7
Group 2	20	171.5	3.8	175.4	162.2	0.8

Mean, mean value; SD, standard deviation; Max, maximum value; Min, minimum value; SE, standard error; Group 1—the 20 of the largest body weight yaks in Gannan yak; Group 2—the 20 of the smallest weight yaks in Gannan yak.

### 2.2 Genomic DNA extraction, resequencing and SNP detection

DNA was extracted from blood samples using the EasyPure Blood Genome DNA Kit (Quanjin, Beijing). Concentration was measured with a Qubit fluorometer (Invitrogen, United States), and integrity was checked via agarose gel electrophoresis. DNA was fragmented to 300–500 bp, followed by terminal repair, 3′-adenylation, adapter ligation, and PCR amplification. The single-stranded PCR product was cyclized, uncyclized DNA was digested, and rolling cycle amplification produced DNA nanoballs (DNB). DNBs were loaded onto nanoarrays and sequenced on the DNBSEQ-T7 platform (BGI, China). Sequencing reads were stored in FASTQ format after base calling analysis. The reads were mapped to the yak reference genome (Bos_grunniens.LU_Bosgru_v3.0) from Ensemble using the Sentieon (12) software (V202112). Sentieon was used to detect the mutation sites of each sample to obtain the gVCF of each sample. Sentieon was used for joint-calling, and the gVCF of all samples was analyzed jointly to obtain the variation results of each individual in the population. To ensure the accuracy of SNP, preliminary hard filtering was performed on the SNP loci obtained after joint analysis (SNP hard filtering criteria: “QD 60.0 | | MQ 3.0 | | MQRankSum <−12.5 | | ReadPosRankSum <−8.0”). To prevent the influence of gender on subsequent analysis and reduce the SNP call errors caused by error mapping or insertion and deletion (InDels), VCFtools (v.0.1.16) (13) was used to extract 1–29 autosomes. Only high-quality SNPs (coverage depth ≥3, RMS mapping quality ≥20, maf ≥0.05, missed ≤0.2) were selected for subsequent analysis.

### 2.3 Population structure and genetic analysis

A principal component analysis (PCA) and runs of homozygosity analysis were conducted using PLINK (v1.07) (14). ROH were identified for estimating homozygosity using PLINK (--homozyg-density 50 --homozyg-gap 100 --homozyg-kb 300 --homozyg-snp 50 --homozyg-window-het 3 --homozyg-window-snp 50 --homozyg-window-threshold 0.05). VCFtools was used to estimate nucleotide diversity in each group with the parameter “--window-pi 50000 --window-pi-step 5000.” Linkage disequilibrium decay with physical distance between SNPs was calculated and visualized by using PopLDdecay (v3.42) (15). The range of gene annotation was determined based on the *r*^2^ value of LD decay (*r*^2^ ≈ 0.1) of 247 Gannan yaks.

### 2.4 Genome-wide association study

The FarmCPU model implemented in the rMVP (v1.2.0) (16) package of the R was utilized to identify SNPs significantly associated with BW phenotypic traits. Quality control was performed on the SNP data, and the genome-wide significance threshold was set to 1/*N*snp, where *N*snp represents the total number of SNPs remaining after quality control. Correction for multiple testing was performed using the Bonferroni method. FarmCPU is a mixed linear model-based approach that alternates between fixed and random effects to balance the detection of true associations and control for confounding factors. In this study, PCA was incorporated as fixed effects to adjust for population stratification, while kinship, calculated automatically by rMVP, was included as random effects to account for genetic relatedness among individuals.

The model is expressed as follows:


(1) $$\begin{array}{l} \mathrm{yi}=\mathrm{Mi}1b1+\mathrm{Mi}2b2+\ldots +\mathrm{Mi}1b1+\mathrm{Mi}2b2yi\\ =\mathrm{Mi}1b1+\mathrm{Mi}2b2+\ldots +\text{Minbn}+\text{Zijuj}+\mathrm{ei} \end{array}$$

(2) yi=Vi+eiyi=Vi+ei

In Equations 1, 2, Equation 1 is a fixed-effect model, Equation 2 is a random-effects model, *y*𝑖 represents the phenotypic observation of the 𝑖-th individual, 𝑀𝑖𝑛 represents the classification result of a total of 𝑛 potential correlation sites included in the model, 𝑏𝑛 indicates the effect value corresponding to the site, 𝑍𝑖𝑗 indicates the classification result of the 𝑗-th marker of the 𝑖-th individual, 𝑢𝑗 is the effect value of 𝑍𝑖𝑗, 𝑉𝑖 represents the total genetic effect of the 𝑖-th individual, and 𝑒𝑖 is the residual vector, subject to 𝑒 ~ 𝑁(0, 𝐼𝜎^2^𝑒)*e* ~ *N*0, *Iσe*^2^. When executing the FarmCPU model, styles Equations 1, 2 are alternate operations. The alternating operation between Equation 1 (fixed-effect model) and Equation 2 (random-effect model) ensures the detection of SNPs associated with phenotypic traits while controlling for population structure and genetic background. By incorporating PCA and kinship, the FarmCPU model effectively minimizes false positives and improves the accuracy of GWAS results.

### 2.5 Genome-wide scanning

Twenty individuals from 247 54-month-old female Gannan yaks with the largest BW were grouped into Group 1, and the 20 individuals also from 247 54-month-old female Gannan yaks with the smallest BW were grouped into Group 2. A *t*-test was conducted to assess whether there was a significant difference in BW between the two groups. We performed genome-wide scanning of Group 1 and Group 2 using *F*_st_ and *θ*_π_-ratio. *F*_st_ values and *θ*_π_ ratios were calculated using 50-kb windows with 5-kb sliding steps in VCFtools. For both metrics, windows in the top 1% were deemed significant. Regions where top 1% windows of *F*_st_ and *θ*_π_-ratio overlapped were identified as candidate selection regions.

### 2.6 Candidate gene analysis

In this study, the yak 3.0 reference genome (Bos_grunniens.LU_Bosgru_v3.0) in Ensmble was used for gene annotation. The gene annotation software was BEDTools (v2.31.1) (17). The range of genes annotated for the significant GWAS site is determined based on the LD decay *r*^2^ value (*r*^2^ ≈ 0.1) in Gannan yaks. The genes for the genome-wide scan are annotated based on the 1% common window range. Venny 2.1.0^1^ was used for the Venn diagram. Metascape^2^ was used for the biological function analyses. The VCFtools was used to calculate the nucleotide diversity in the target region with 5-kb windows and to extract haplotypes. The PLINK software was used to calculate the LD.

## 3 Results

### 3.1 Genetic diversity analysis of yak

**Figure 2:**
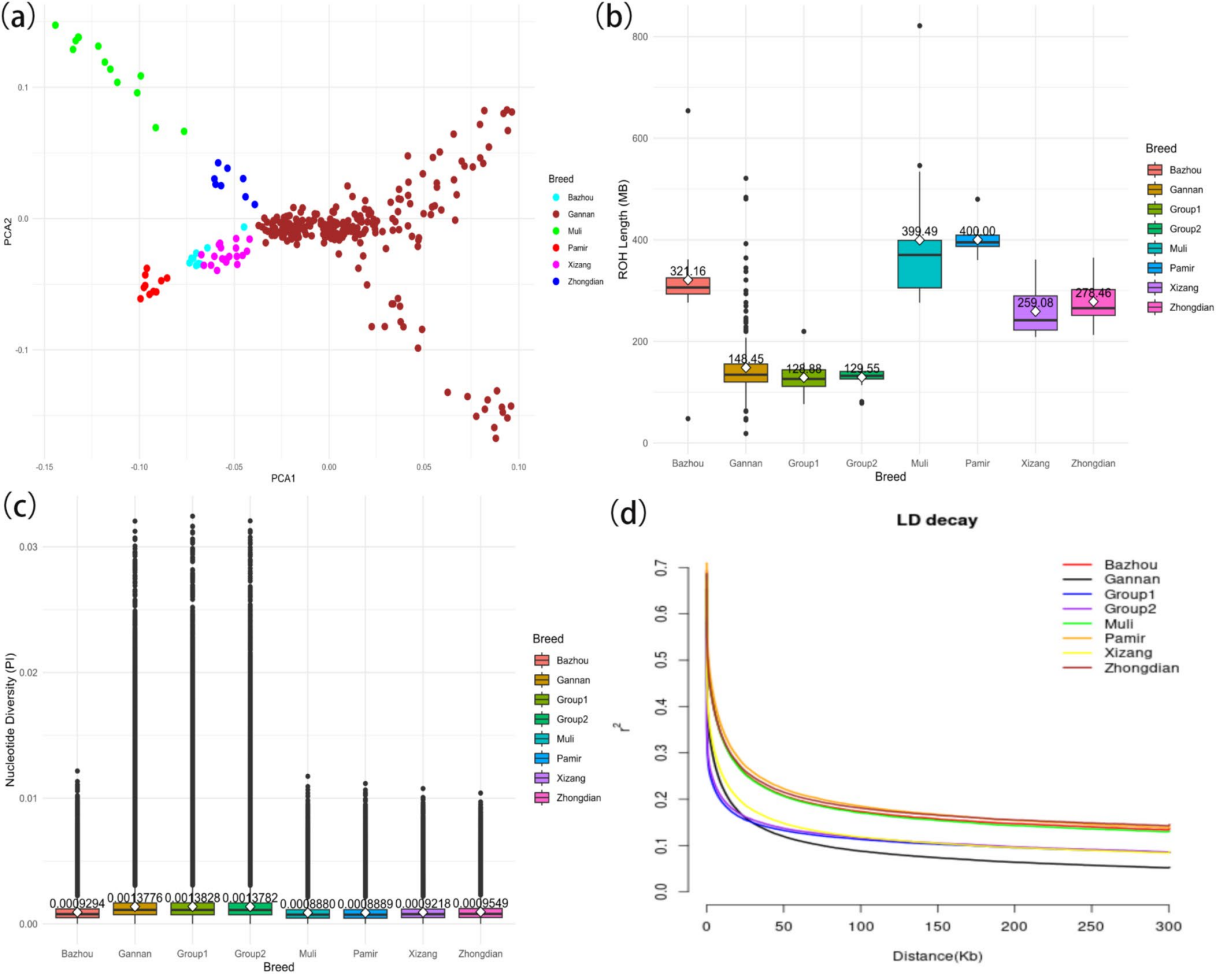
Summary statistics of principal components and genomic variation of six yak breeds. **(A)** Principal component analysis of six yak breeds. **(B)** The ROH of yaks. **(C)** The nucleotide diversity of yaks. The horizontal line within the box denotes the median value, whereas the boundaries of the box represent the first and third quartiles. Outliers are represented as individual points. The average is the white dot on the line, and the number of the average is also indicated above the white dot. **(D)** Linkage disequilibrium decay of yaks.

This study examined the genetic diversity of 309 yaks from six distinct breeds. Principal component analysis (PCA) revealed clear genetic differentiation between Gannan yaks and the other five breeds (Figure 2A). Notably, Gannan yaks exhibited shorter runs of homozygosity (ROH) segments compared to Pamir yaks, indicating lower recent inbreeding levels (Figure 2B). Additionally, the ROH of Group 1 yaks was shorter than that of Group 2 yaks, suggesting a greater genetic diversity in the former. Nucleotide diversity analyses highlighted that Gannan yaks had the highest diversity among the breeds, contrasting with the lowest values observed in Muli yaks (Figure 2C). Furthermore, Group 1 demonstrated higher nucleotide diversity compared to Group 2, reflecting greater genetic variation in larger individuals. Linkage disequilibrium (LD) decay analysis showed that Gannan yaks exhibited the fastest decay rates, indicating a higher recombination rate or older population structure (Figure 2D). These findings collectively underscore the rich genetic diversity and unique evolutionary history of Gannan yaks, with Group 1 displaying greater variation than Group 2.

### 3.2 Statistics of Gannan yak body weight

**Figure 3:**
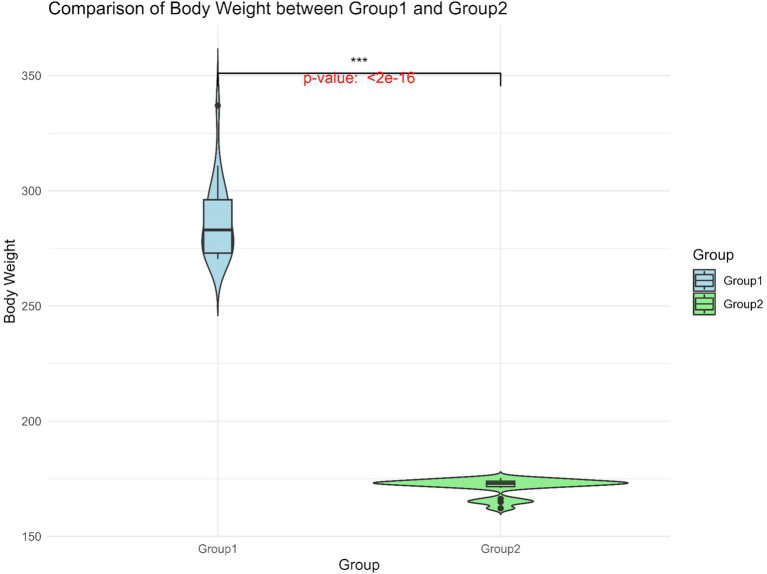
Body weight distribution of Group 1 and Group 2. The horizontal line within the box denotes the median value, whereas the boundaries of the box represent the first and third quartiles. Outliers are represented as individual points. ^***^Significant at *p* < 0.001.

BW data for 247 Gannan yaks were summarized (Table 1). The overall mean BW was 218.0 ± 34.6 kg, with Group 1 exhibiting significantly higher BW (287.0 ± 16.6 kg) compared to Group 2 (171.5 ± 3.8 kg) (Figure 3). The large standard deviation and range reflect the extensive phenotypic variation within the population. This significant BW difference between the groups provided a basis for identifying genetic factors underlying this trait.

### 3.3 Genotype calling and analyzing of 247 Gannan yaks

**Figure 4:**
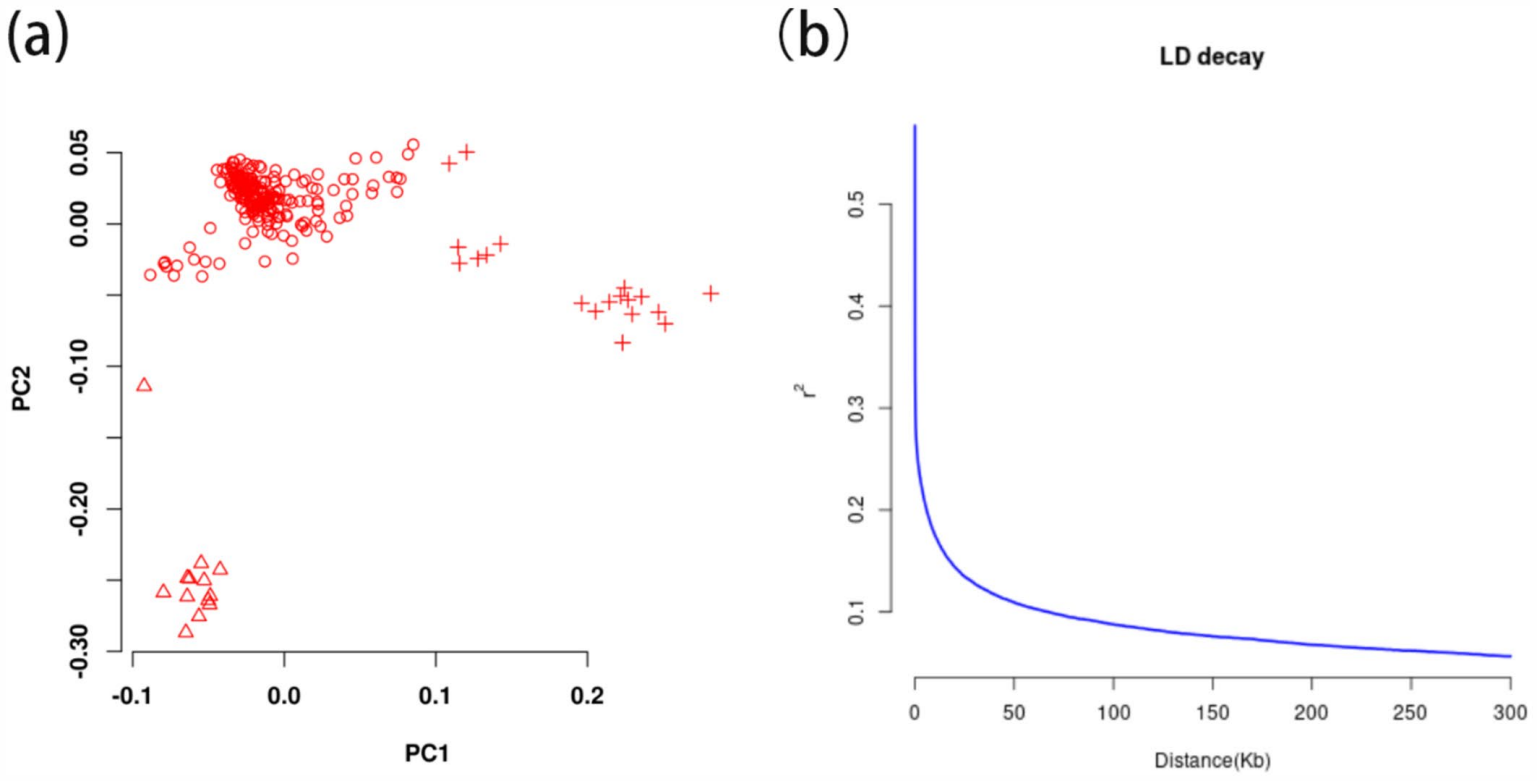
The principal component analysis and linkage disequilibrium decay calculation of 247 Gannan yak. **(A)** Principal components analysis of 247 Gannan yaks. Different symbols represent the grouping situation. **(B)** Linkage disequilibrium decay of 247 Gannan yaks.

After filtering, 12411513 valid high-quality SNPs of 247 Ganan yaks were retained. The PCA1 and PCA2 divided 247 Gannan yaks into three groups in Figure 4A, demonstrating stratification among the Gannan yaks. As a result, PCA = 3 was incorporated into the FarmCPU model for adjustment, and the kinship file generated during the rMVP analysis was also included in the FarmCPU model to improve the accuracy of GWAS. LD analysis revealed that LD decayed to a stable level (*r*^2^ ≈ 0.1) within 50–60 kb, indicating that regions within this range were suitable for candidate gene annotation (Figure 4B). Therefore, we selected a ±50 kb window around significant GWAS site as the annotation range for candidate genes.

### 3.4 GWAS analysis results

**Figure 5:**
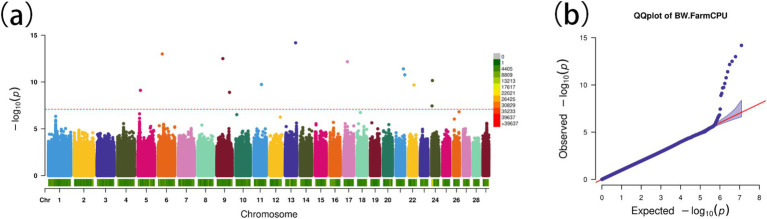
Genome-wide association study for body weight in Gannan yaks. **(A)** Manhattan map of genome-wide association study. **(B)** Quantile–Quantile plots from the genome-wide association study.

Using the FarmCPU model, 13 significant SNPs associated with BW were identified (Figures 5A,B). These SNPs annotated 19 genes (Table 2), including several with known roles in growth and metabolism. For example, *LRPPRC* is involved in cytoskeletal regulation and bone development, *PMAIP1* is associated with obesity, and *PPP1R11* has roles in cell proliferation and fat metabolism. Additionally, *GABBR1*, involved in fatty acid metabolism, emerged as a novel candidate. These genes provide insights into the molecular mechanisms influencing BW in Gannan yaks.

**Table 2:** The information on candidate genes from the genome-wide association study.

Chromosome	Position	*p*-value	Gene name	Gene position
6	31311271	7.88 × 10^−7^	*BTC*	31225858–31278471
9	46247284	3.19 × 10^−13^	*LRPPRC*	46152562–46258899
9	97711609	1.29 × 10^−13^	*OTOF*	97665582–97696329
9	97711609	1.29 × 10^−13^	*DRC1*	97697741–97740010
9	97711609	1.29 × 10^−13^	*SELENOI*	97753607–97797598
11	58069337	1.86 × 10^−10^	*OTX2*	57983921–57991821
13	80629523	6.56 × 10^−15^	*CNTN5*	79939901–80974967
17	28641668	6.87 × 10^−13^	*TMC3*	28497725–28556487
21	57816348	4.02 × 10^−12^	*PMAIP1*	57811222–57813908
24	3313732	3.63 × 10^−8^	*H2BC6*	3223813–3224193
24	3313732	3.36 × 10^−8^	*H2AC8*	3248645–3249037
24	3313732	3.36 × 10^−8^	*H4C8*	3319662–3319973
24	3313732	3.36 × 10^−8^	*BTN2A2*	3411192–3422262
24	6221834	7.02 × 10^−11^	*POLR1H*	6226933–6230864
24	6221834	7.02 × 10^−11^	*PPP1R11*	6235327–6237209
24	6221834	7.02 × 10^−11^	*MOG*	6188533–6198769
24	6221834	7.02 × 10^−11^	*RNF39*	6241382–6246935
24	6221834	7.02 × 10^−11^	*TRIM40*	6318264–6330272
24	6221834	7.02 × 10^−11^	*GABBR1*	6134269–6164856

### 3.5 The result of genome-wide scanning

**Figure 6:**
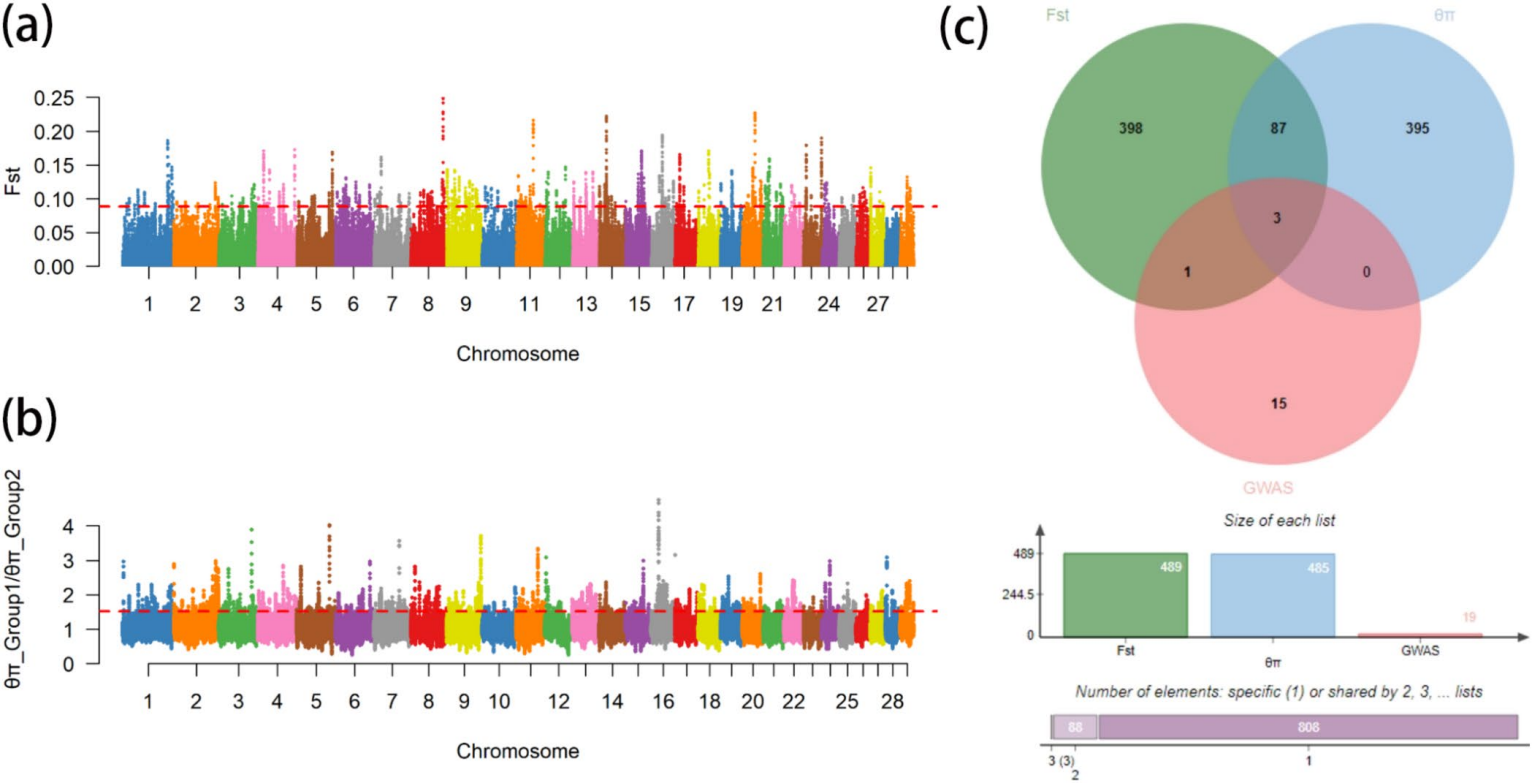
Genome-wide scanning of body weight in Gannan yaks. **(A)** Manhattan map of *F*_st_. **(B)** Manhattan map of *θ*_π_-ratio. **(C)** Gene numbers of three methods.

**Figure 7:**
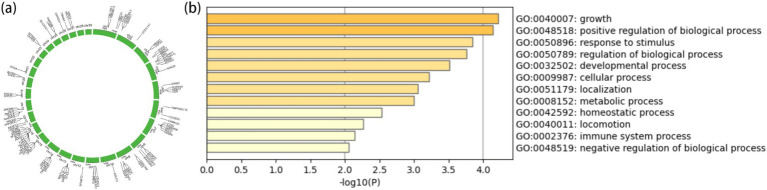
Gene annotation and enrichment analysis. **(A)** The top 1% windows overlapping genes of *F*_st_ and *θ*_π_-ratio. **(B)** Enrichment analysis of top 1% windows overlapping genes (*p* ≤ 0.05).

From the results of Figures 2, 3, the genotype and phenotype of Group 1 are different from Group 2. To further explore the genes associated with BW, we searched the whole genomic region of Group 1 and Group 2 using two statistical methods (*F*_st_ and *θ*_π_-ratio) to compare the Group 1 and Group 2 based on sliding 50-kb windows with 5-kb steps (Figures 6A,B). The regions within the top 1% windows were considered candidate genomic regions. We identified 489 and 485 candidate genes through *F*_st_ and *θ*_π_-ratio (Figure 6C). On combining *F*_st_ and *θ*_π_-ratio analyses, we identified 90 overlapping genes (Figures 6C, 7A). To enhance the understanding of the functions associated with overlapping genes, an enrichment analysis was conducted using Metascape. Among them, *FGF2*, *SHH* and *WNT11* were enriched in five Gene Ontology biological processes: growth, positive regulation of biological process, regulation of biological process, developmental process and metabolic process (Figure 7B). *OTOF*, *DRC1*, and *SELENOI* overlap in the GWAS and the genome-wide scanning, prompting more comprehensive future studies.

### 3.6 Gene analysis

**Figure 8:**
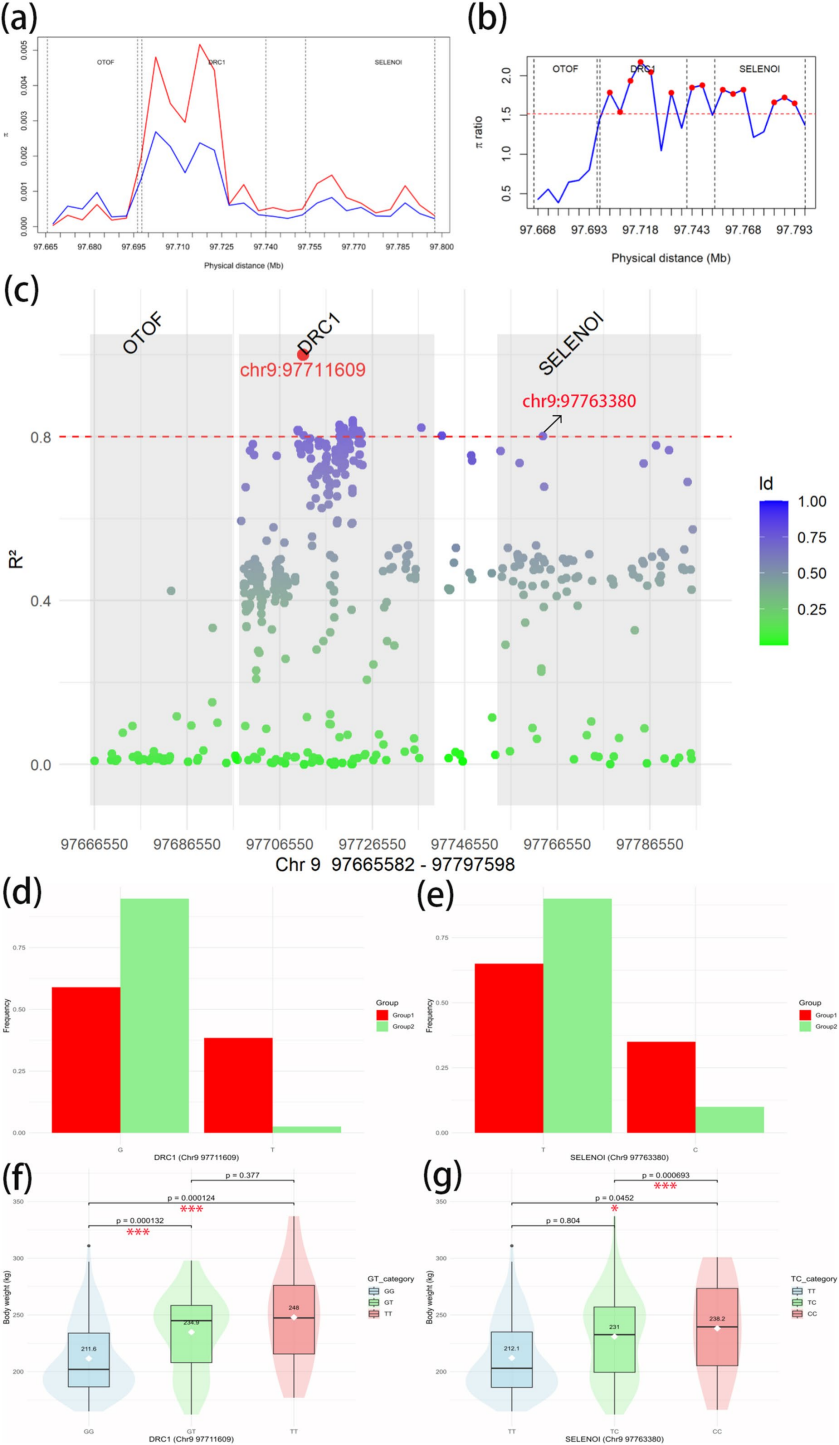
Candidate gene region analysis. **(A)** Nucleotide diversity in *Chr9 97665582*–*97797598* regions. Red represents the Group 1; blue represents the Group 2. **(B)** Nucleotide diversity ratio (Group 1/Group 2) in *Chr9 97665582*–*97797598*. The red dashed line is the threshold line for the top 1% window of the whole-genome scan *θ*_π_-ratio. Red dots indicate windows above the threshold line. The vertical dotted lines in the figure are the dividing lines between genes. **(C)** Linkage imbalance with significant GWAS site *Chr9 97711609* to other SNPs in *Chr9 97665582*–*97797598* regions of 247 Gannan yaks. The dotted red line is the strong linkage threshold line of *R*^2^ = 0.8. The gray box shows the range of each of the three genes. **(D)** The allele frequencies of *G* and *T* at *Chr9 97711603* site in Group 1 and Group 2. *G* is the wild-type and *T* is the mutant-type. **(E)** The allele frequencies of *T* and *C* at *Chr9 97763380* site in Group 1 and Group 2. *T* is the wild-type and *C* is the mutant-type. **(F)** Body weight phenotypic differences between haplotypes of *DRC1* in *Chr9 97711603* site. **(G)** Body weight phenotypic differences between haplotypes of *SELENOI* in *Chr9 97763380* site. ^*^Significant at *p* < 0.05, ^**^significant at *p* < 0.01, and ^***^significant at *p* < 0.001.

To further investigate the association between the overlapping genes *OTOF*, *DRC1*, and *SELENOI* and BW in Gannan yaks, we calculated the nucleotide diversity and nucleotide diversity ratio between Group 1 and Group 2 in *Chr9 97665582*–*97797598* regions (Figures 8A,B). In *DRC1* and *SELENOI*, the nucleotide diversity in most windows of Group 1 was higher than that of Group 2. After that, we calculated the LD values of the significant GWAS site (*Chr9 97711609*) in the *Chr9 97665582*–*97797598* region (Figure 8C), the results showed that there was no *R^2^* ≥ 0.8 SNPs were located in the *OTOF* gene, meanwhile, the *Chr9 97763380* (*R*^2^ > 0.8) site fell inside the *SELENOI* gene. It indicated that *DRC1* and *SELENOI* were more likely to be the candidate genes for BW of Gannan yak. To further prove that *DRC1* and *SELENOI* are candidate genes for BW, we selected two sites (*Chr9 97711609* and *Chr9 97763380*) for analysis. The allele frequency showed that most of the mutant-type (*Chr9 97711069* for *T*, *Chr9 97763380* for *C*) of the two sites were concentrated in Group 1, while most of the wild-type (*Chr9 97711069* for *G*, *Chr9 97763380* for *T*) were concentrated in Group 2 (Figures 8D,E). This can also prove to a certain extent that the variation of Group 1 is richer than that of Group 2, and also shows that the mutation of DRC1 (*T* genotype of *Chr9 97711609*) and the mutation of *SELENOI* (*C* genotype of *Chr9 97711609*) may be associated with BW. Finally, all haplotypes of 247 Gannan yaks at these two sites were extracted, and the difference testing was carried out according to different haplotype groups combined with BW data (Figures 8E,F). The results showed that the mean BW of homozygous mutant-type (*TT* of *DRC1* and *CC* of *SELENOI*) was the largest. The mean BW of homozygous wild-type (*GG* of *DRC1* and *TT* of *SELENOI*) was the smallest. The difference in BW between the two groups was significant (Figures 8F,G), reinforcing the association of *DRC1* and *SELENOI* mutations with BW in Gannan yaks.

## 4 Discussion

The genetic diversity and BW traits of Gannan yaks hold significant implications for their breeding and conservation. Historically, Gannan yaks have been managed under mixed grazing systems in natural grasslands with limited selective breeding efforts. Unlike modern livestock species such as beef cattle, where systematic breeding programs have achieved substantial genetic improvement, yak breeding has faced challenges due to a lack of pedigree records, reliance on traditional management practices, and limited technological intervention. This study’s findings offer valuable insights into the genetic underpinnings of BW in Gannan yaks and provide a molecular basis for advancing their breeding programs.

ROH lengths offer insight into inbreeding history: shorter ROHs indicate older inbreeding, while longer ROHs point to more recent, closer inbreeding events (18). This study revealed that Gannan yaks possess higher genetic diversity compared to other yak breeds, as indicated by the shortest runs of homozygosity (ROH) and the fastest LD decay rates. These findings suggest that Gannan yaks have experienced less recent inbreeding and maintain a relatively large effective population size. Within the Gannan yak population, the high BW group (Group 1) demonstrated even greater genetic diversity than the low BW group (Group 2). This variability highlights the potential of utilizing existing genetic resources within the breed to improve growth traits through selective breeding. The identification of high genetic diversity in Gannan yaks aligns with their historical reliance on natural grazing, which has likely preserved genetic variation due to the absence of intensive artificial selection. However, this diversity also reflects the underdeveloped state of yak breeding programs. Systematic efforts to harness this genetic diversity through marker-assisted selection and GWAS could enhance the productivity of Gannan yaks while retaining their adaptability to the harsh plateau environment.

The study identified 19 genes associated with BW through GWAS, with notable candidates including *LRPPRC*, *PMAIP1*, *PPP1R11*, and *GABBR1*. These genes are involved in critical biological processes such as muscle development, fat metabolism, and cell proliferation. *LRPPRC* plays a role in regulating cytoskeletal network dynamics (19), It influences conformation traits in Danish pig breeds by modulating bone and muscle development (20). *PMAIP1* has been linked to human obesity (21). *PPP1R11* encodes phosphatase 1 regulatory (inhibitor) subunit 11, it may be involved in Hippo signaling in apoptosis and cell proliferation and has been linked to *VLDL* particle concentration in plasma (22, 23). *GABBR1*, which encodes a receptor for gamma-aminobutyric acid (GABA) involved in the cAMP signaling pathway, plays a role in fatty acid β-oxidation and degradation. This gene has also been linked to fat depot-specific adipose tissue macrophage infiltration in obesity (24). These findings suggest that the genetic architecture of BW in Gannan yaks is influenced by pathways that overlap with those in other livestock species, providing an opportunity to leverage cross-species knowledge for yak breeding.

The genome-wide scanning analysis further identified 90 candidate genes, including *FGF2*, *SHH* and *WNT11*, which are enriched in growth and developmental processes. *SHH* (*Sonic Hedgehog*) promotes the growth of intestinal epithelium by activating the SHH-WNT signaling axis (25). During the morphological changes occurring in osteoblast differentiation, the epigenetic machinery in modulating the *BMP7*-induced osteogenic phenotype by influencing the activity of *Shh-related* genes (26). Shh-induced Akt phosphorylation promotes muscle cell proliferation and differentiation, and *SHH* is critical for controlling specific ventral muscle formation (27, 28). Fibroblast growth factor 2 (*FGF2*) is a potent mitogenic growth factor expressed in limb bud mesenchyme, chondrocytes, osteoblasts, osteoclasts, and adipocytes, promoting osteoblast differentiation by regulating *BMP2* and *ATF4* as well as Wnt/β-catenin pathway (29, 30). *FGF2-null* mice exhibit osteopenia and reduced bone remodeling (31–33). In addition, N-acetylated *HS* mimics promote *FGF2* signaling, thereby promoting muscle stem cell expansion (34). *WNT11* acts as a directional cue to organize myotube elongation in the myotome via the noncanonical Wnt/planar cell polarity pathway (35), co-expression of *Akt1* and *Wnt11* significantly boosts the proliferation and growth of mesenchymal stem cells, while Wnt11 overexpression synergistically promotes chondrogenic differentiation with TGF-β (36, 37). These genes underscore the complex genetic regulation of BW and highlight targets for future functional studies.

Through the analysis of the three overlapping genes, *DRC1* and *SELENOI* were identified as key candidate genes for BW in Gannan yak. Other studies demonstrated that *DRC1* is a critical gene in cell cycle-regulated essential for DNA replication and necessary for the S-phase checkpoint, including the proper activation of Rad53 kinase in response to DNA damage and replication interruptions (38). Furthermore, *DRC1* expression in follicular dendritic cells (FDCs) underscores its association with muscle cells. FDCs expressing *DRC1* also express α-smooth muscle actin (α-SM actin) and form contractile stress fibers, key features of muscle cells, particularly myofibroblasts (39). *SELENOI* plays a role in maintaining redox-related calcium homeostasis and helps mitigate muscle degeneration and related kyphosis (40–42). Its expression is also upregulated in the skeletal muscles of chickens in response to selenium deficiency in the diet (43). These findings indicated that *DRC1* and *SELENOI* play roles in DNA replication, muscle cell function, and metabolic homeostasis, making them promising targets for improving BW in Gannan yaks.

The identification of candidate genes for BW provides a scientific foundation for addressing long-standing challenges in Gannan yak breeding. Unlike beef cattle, where breeding programs have been optimized for traits such as growth rate and feed efficiency, Gannan yak breeding has lagged due to limited genomic resources and infrastructure. This study bridges this gap by identifying molecular markers that can be integrated into selective breeding programs. Furthermore, the high genetic diversity observed in Gannan yaks suggests that *in situ* conservation strategies can be complemented by genetic improvement efforts. By prioritizing the identified candidate genes in breeding programs, it is possible to enhance BW traits without compromising the genetic diversity that underpins the breed’s adaptability. The findings also provide a framework for developing genomic selection models tailored to the unique characteristics of Gannan yaks, accelerating the pace of genetic improvement.

This study demonstrates the utility of combining GWAS, genome-wide scanning, functional annotation and SNP association analyses to dissect complex traits in livestock species with limited breeding histories. The findings highlight the potential of genomic tools to revolutionize yak breeding, moving beyond traditional practices to data-driven approaches. However, realizing these benefits will require sustained investment in genomic infrastructure, farmer education, and policy support. Future research should focus on validating the functional roles of the identified genes and exploring gene-environment interactions that may influence BW. Additionally, integrating genomic data with phenotypic records and environmental variables could provide a holistic understanding of the factors shaping BW traits in Gannan yaks. These efforts will be essential for ensuring the sustainability and competitiveness of yak husbandry in the face of changing environmental and market conditions.

In conclusion, this study lays the groundwork for transforming Gannan yak breeding through genomic insights. By leveraging the genetic diversity and candidate genes identified here, it is possible to enhance the economic and cultural value of this iconic breed while preserving its ecological resilience.

## Data Availability

The original contributions presented in the study are publicly available. These data can be found in https://doi.org/10.6084/m9.figshare.29143589.

## Footnotes

^1^ https://bioinfogp.cnb.csic.es/tools/venny/index.html

^2^ https://metascape.org/gp/index.html
